# Rhamnose-Containing Compounds: Biosynthesis and Applications

**DOI:** 10.3390/molecules27165315

**Published:** 2022-08-20

**Authors:** Siqiang Li, Fujia Chen, Yun Li, Lizhen Wang, Hongyan Li, Guofeng Gu, Enzhong Li

**Affiliations:** 1 School of Biological and Food Processing Engineering, Huanghuai University, Zhumadian 463000, China; 2 Institute of Agricultural Products Fermentation Engineering and Application, Huanghuai University, Zhumadian 463000, China; 3 Biology Institute, Qilu University of Technology (Shandong Academy of Sciences), Jinan 250100, China; 4 National Glycoengineering Research Center, Shandong Key Laboratory of Carbohydrate Chemistry and Glycobiology, Shandong University, 72 Binhai Road, Qingdao 266237, China

**Keywords:** rhamnose, deoxythymidinediphosphate-L-rhamnose, guanosine diphosphate rhamnose, uridine diphosphate-rhamnose, rhamnosyltransferase, rhamnose biosynthesis

## Abstract

Rhamnose-associated molecules are attracting attention because they are present in bacteria but not mammals, making them potentially useful as antibacterial agents. Additionally, they are also valuable for tumor immunotherapy. Thus, studies on the functions and biosynthetic pathways of rhamnose-containing compounds are in progress. In this paper, studies on the biosynthetic pathways of three rhamnose donors, i.e., deoxythymidinediphosphate-L-rhamnose (dTDP-Rha), uridine diphosphate-rhamnose (UDP-Rha), and guanosine diphosphate rhamnose (GDP-Rha), are firstly reviewed, together with the functions and crystal structures of those associated enzymes. Among them, dTDP-Rha is the most common rhamnose donor, and four enzymes, including glucose-1-phosphate thymidylyltransferase RmlA, dTDP-Glc-4,6-dehydratase RmlB, dTDP-4-keto-6-deoxy-Glc-3,5-epimerase RmlC, and dTDP-4-keto-Rha reductase RmlD, are involved in its biosynthesis. Secondly, several known rhamnosyltransferases from *Geobacillus stearothermophilus*, *Saccharopolyspora spinosa*, *Mycobacterium tuberculosis*, *Pseudomonas aeruginosa*, and *Streptococcus pneumoniae* are discussed. In these studies, however, the functions of rhamnosyltransferases were verified by employing gene knockout and radiolabeled substrates, which were almost impossible to obtain and characterize the products of enzymatic reactions. Finally, the application of rhamnose-containing compounds in disease treatments is briefly described.

## 1. Introduction

Glycans are important components of various glycoconjugates, such as glycoproteins, glycolipids, and proteoglycans, and play pivotal roles in many biological processes, including intracellular trafficking, cell adhesion and development, cancer progression, host–pathogen interactions, and immune responses [1]. For a detailed structure–activity relationship analysis of functional glycans, it is necessary to obtain molecules in structurally homogeneous forms, which is not easy to achieve via the isolation of natural products from biological sources. Therefore, the total synthesis of polysaccharides and their oligomeric analogs has become a hot research topic. Rhamnose (Rha)-containing compounds (RCCs) are especially interesting due to their potential applications, including antibacterial vaccines and killing tumors [2,3]. Additionally, Rha is a common component of various bacterial polysaccharides, such as lipopolysaccharides (LPSs) [4], extracellular polysaccharides (EPSs) [5], capsular polysaccharides (CPSs) [6], and cell wall polysaccharides [7]. In addition to bacteria, Rha is also found in viruses [8], fungi [9], plants [10], and lower animals [11]. Interestingly, Rha has not been found in humans or other mammals. In recent years, more evidence has emerged about its essential roles in many pathogenic bacteria, making it a potentially attractive therapeutic target. Furthermore, RCCs are also candidates for vaccines, antitumor drugs, and antibacterial drugs [3,12]. Thus, there is a keen desire to obtain and characterize RCCs. However, due to the complexity of the target molecules and the difficulty in constructing certain glycosidic linkages, such as β-linked Rha, via chemical glycosylation, enzymatic synthesis is particularly attractive [13]. For the enzymatic synthesis of RCCs or their conjugates, rhamnosyl donors are the key substrates [14,15], which are utilized by rhamnosyltransferases (Rha-Ts) and attached to sugar acceptors [16]. Therefore, the catalysis of Rha-Ts and the preparation of Rha donors and acceptors in vitro are hot topics [13,17,18]. In this paper, the biosynthetic pathways of Rha donors are reviewed, and the development of Rha-Ts and their medical perspectives are also explored. Such knowledge expands our understandings of the biosynthetic pathways of RCCs and could facilitate their enzymatic synthesis.

## 2. Biosynthetic Pathways of Donors of RCCs

Three sugar nucleotides, including deoxythymidinediphosphate-L-rhamnose (dTDP-Rha), guanosine diphosphate rhamnose (GDP-Rha), and uridine diphosphate-rhamnose (UDP-Rha), can serve as Rha donors in reactions catalyzed by Rha-Ts. dTDP-Rha and GDP-Rha are present in bacteria and fungi, whereas UDP-Rha is only found in plants. There are probably other Rha donors involving in Rha biosynthetic pathways in *Mycoplasma* [19]. The biosynthetic pathways of these three Rha donors and structural, mechanistic, and biochemical aspects of the key enzymes involved are reviewed below.

### 2.1. Biosynthetic Pathways of dTDP-Rha

**Figure 1:**
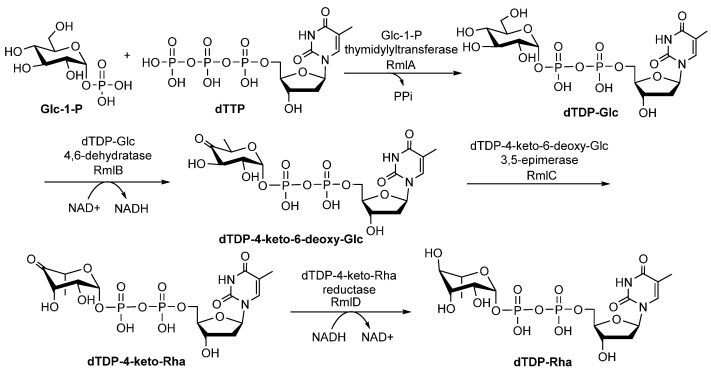
The biosynthetic pathway of dTDP-Rha from Glc-1-P in bacteria [22,23].

The dTDP-Rha is one of the most important sugar precursors. Four enzymes, glucose-1-phosphate thymidylyltransferase (RmlA), dTDP-D-glucose 4,6-dehydratase (RmlB), dTDP-4-keto-6-deoxy-D-glucose3,5-epimerase (RmlC), and dTDP-4-keto-L-Rha reductase (RmlD), are responsible for the formation of dTDP-Rha (Figure 1) [20]. The *rmlA*, *rmlB*, *rmlC*, and *rmlD* genes are usually located in biosynthetic gene clusters of polysaccharides in conserved gene orders with few exceptions [20]. Below, we discuss what is known about the steps involved in the biosynthesis of dTDP-Rha, as well as the functions, physicochemical properties, and crystal structures of RmlA, RmlB, RmlC, and RmlD [21].

**Figure 2:**
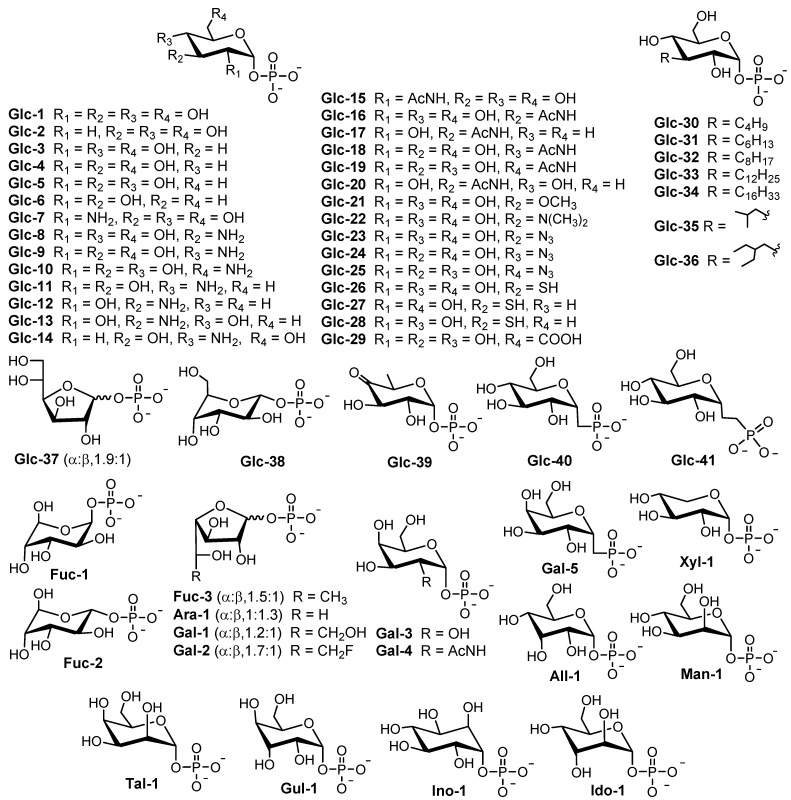
Sugar-1-phosphates recognized by RmlA [22].

RmlA is a nucleotidyltransferase that catalyzes the first reaction to form dTDP-glucose (dTDP-Glc) by transferring a deoxythymidine triphosphate (dTTP) to glucose-1-phosphate (Glc-1-P) via a single sequential displacement mechanism [24]. Based on the reverse reaction, RmlA is also known to be a pyrophosphorylase [24]. RmlA has attracted more attention because it displays unusual promiscuity toward both sugar-1-phosphates and nucleotide triphosphate substrates, which could be harnessed in glycorandomization [25,26]. The inherent sugar-1-phosphate and/or nucleotide triphosphate (NTP) promiscuity of RmlA was further expanded by mutation studies. For example, L89T [27], E162D, Y177F [27], T201A, and W224H [28] mutants increased its sugar-1-phosphate tolerance and conversion [27], whereas Q24S [29] and Q83D/S [30] mutants altered the preference for the NTP of wildtype RmlA (also called the inherent NTP purine/pyrimidine bias). RmlA and its variants can utilize 57 sugar-1-phosphates ranging from all epimers [26], substituted compounds (amino [30], N-acetyl [31], methyl [32], azido [32], thiol [32], and alkyl [33]), deoxy sugars of D-glucose [32], two anomers of L-fucose [34], to pentofuranosyl-1-phosphate [34]. In addition to sugar-1-phosphate substrates, Moretti et al. reported that RmlA could recognize all eight natural NTPs as substrates despite its reduced activity toward purine NTPs [35]. Furthermore, Cps2L (a RmlA homolog) can act on deoxythymidine 5-tetraphosphate (p4dT) and Glc-1-P to form dTDP-Glc and triphosphate (PPPi) [32]. To date, 154 (d) nucleotide diphosphate (NDP)-sugars have been produced by RmlA and its variants (Figure 2 and Supplementary information Table S1) [36,37,38,39,40,41,42].

**Figure 3:**
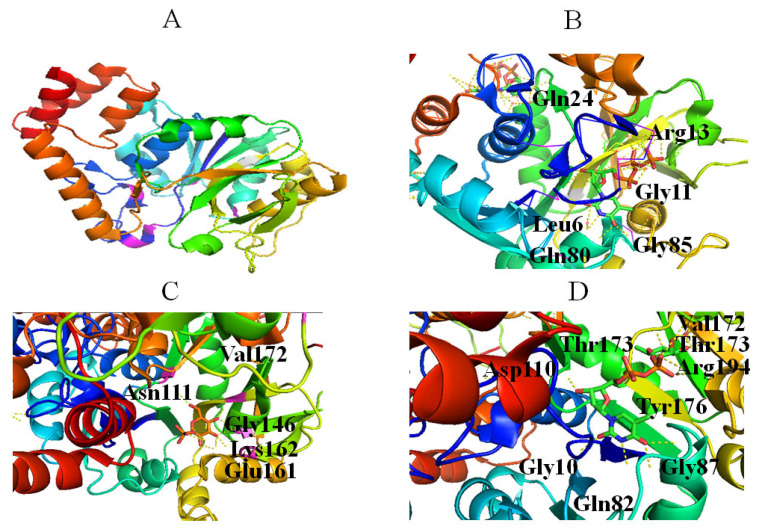
Stereo views of RmlA (Protein Data Bank (PDB) entry 4HO3) (**A**), dTTP (PDB entry 4HO3) (**B**), Glc-1-P (PDB entry 1G23) (**C**), and dTDP-Rha (PDB entry 1G3L) (**D**) bound to RmlA. Hydrogen bonds are shown as red lines. Helices, sheets, and loops of RmlA are colored blue, purple, and beige, respectively. C, N, O, and P elements of ligands are shown in green, blue, red, and brown, respectively. RmlA is shown in cartoon representation, and ligands are shown as sticks [22].

The activity of RmlA is inhibited by dTDP-Glc, inorganic pyrophosphate (PPi) and dTDP-Rha [43], and dTDP-Rha is both a competitive and a noncompetitive inhibitor [43]. Although the mechanism of noncompetitive inhibition of RmlA by dTDP-Rha proposed by Mmot et al. remains unclear and needs to be further studied [43], the mechanism of competitive inhibition is well understood [44], which involved: (1) dTDP-Rha occupies the same site as dTDP-Glc; E161 of RmlA interacts with O2′ and O3′ of Rha through a bidentate hydrogen, similar to the dTDP-Glc complex (Figure 3D); (2) The two phosphates of dTDP-Rha move into the active site and form strong salt bridges with R194, which is absent in the dTDP-Glc complex; (3) A hydrogen bond between ribose O3 of dTDP-Rha and the side chain of D110 is likely replaced by the α-phosphate, resulting in the decomposition of dTDP-Rha. Thus, it was concluded that targeting these sites could provide a potential basis for inhibitor design. In addition, the R15 loop probably affects catalytic activity because it is different in the active site of the dTDP-Rha complex [44]. Crystal structures of RmlA from *Pseudomonas aeruginosa* [44], *Escherichia coli* [45], and *Salmonella typhimurium* [45] showed that RmlA is a homotetramer (Figure 3A).

The active center of RmlA lies in a deep pocket formed by core and sugar-binding domains [45]. G11, Q80, and G85 form hydrogen bonds with the thymine: N3 and O4 of thymine engage in hydrogen bonds with Q80; O4 of thymine also forms hydrogen bonds with the N atom of G85; O2 of the thymine base engages in hydrogen bonds with G11 (Figure 3B) [45]. Neither methyl group of the pyrimidine ring nor the 2-OH of ribose interacts with RmlA, which explains why RmlA can accept UTP and dTTP as substrates.

The 3-hydroxyl group of ribose contacts Q24 (Figure 3B) [45], and glucose residue interacts with RmlA via hydrogen bonds. Specifically, O2 and O3 of glucose form hydrogen bonds with E161; O2, O3, and O4 of glucose form hydrogen bonds with G146 and L172; O6 of glucose forms hydrogen bonds with N111 (Figure 3C). In addition, Q26, G11, S13, and two water molecules bind magnesium. Therefore, the crystal structures of RmlA helped to reveal the reaction mechanism and provide a basis for active site engineering of RmlA [35].

**Figure 4:**
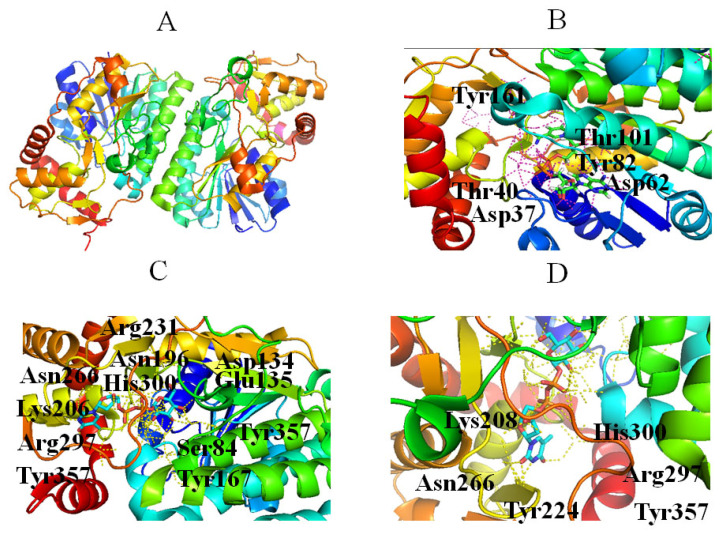
Stereo view of RmlB (PDB entry 1KEP) (**A**), NAD^+^ (PDB entry 1KEP) (**B**), and dTDP-Glc (PDBentry 1KEU) (**C**) bound to RmlB, and the position of the Asn266 side chain as the sugar moves to stack against Tyr224 (**D**). Hydrogen bonds are shown as red lines. Helices, sheets, and loops of RmlB are colored blue, purple, and beige, respectively. C, N, O, and P elements of ligands are green, blue, red, and brown, respectively. RmlB is shown in cartoon representation, and ligands are shown as sticks [22].

The second step in the dTDP-Rha biosynthetic pathway is the dehydration of dTDP-Glc to form dTDP-4-keto-6-deoxy-D-glucose (dT4k6dG), which is catalyzed by RmlB. Four steps have been proposed during the reaction: (1) NAD^+^ extracts a hydride from C4 of the glucose ring; (2) Glu135 removes a C5 proton; (3) elimination of a water molecule between C5 and C6 generates 4-keto-5,6-glucosene as an intermediate; and (4) a hydride is transferred from NADH to C6 of the glucose ring [46]. The substrate tolerance of RmlB is more limited compared with that of RmlA, probably because it catalyzes the committal step in the dTDP-Rha biosynthetic pathway [47]. The crystal structure of RmlB from *Salmonella enterica* serovar Typhimurium showed that it functions as a homodimer (Figure 4A). RmlB has two domains: a larger N-terminal domain consisting of seven β-strands and ten α-helices to bind the nucleotide cofactor NAD^+^; and a smaller C-terminal domain composed of four β-strands and six α-helices to bind dTDP-Glc [46]. The two domains create a deep cavity in the enzyme to form the active site (Figure 4A) [46]. The key residues interacting with NAD^+^ include (1) a hydrogen bond (Asp62) and a hydrophobic crevice consisting of Ile21, Ala57, Ile59, Val77, Ala81, and Leu107 binding to the adenine portion of NAD^+^, and (2) Asp37, Tyr161, and Lys171 forming hydrogen bonds with the ribose sugar (Figure 4B) [46]. In addition, Thr133, Asp134, Glu135, Asn196, Arg231, and Asn266 make contacts with dTDP-Glc (Figure 4C) [46]. Specifically, Thr133, Glu135, and Asp134 bind to the 4, 6-hydroxyl groups of the glucose ring (Figure 4C), while Asn196 and Arg231 interact with the phosphoryl oxygen atom (Figure 4C), and Asn266 hydrogen binds to the 3-hydroxyl group of the ribose sugar (Figure 4D) [46]. Notably, Asn266 may also control the selectivity for the deoxy-nucleotide sugar substrate in the binding site [46].

**Figure 5:**
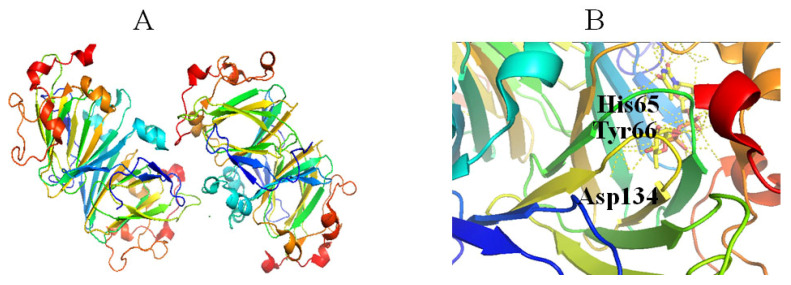
Stereo view of the RmlC (PDB entry 2IXL) (**A**) and His-Asp dyad of RmlC (PDB entry 2IXT) (**B**). Helix, sheet, and loop of RmlC are shown as blue, purple, and beige, respectively. C, N, O, and P elements of ligands are green, blue, red, and brown, respectively. RmlC is shown in cartoon representation, and ligands are shown as sticks [22].

RmlC catalyzes the third step in the dTDP-Rha biosynthetic pathway, in which the C3 and C5 positions of dT4k6dG are epimerized to generate dTDP-4-keto-Rha [48]. The catalytic mechanism of this catalytic reaction is proposed as follows: (1) a proton is abstracted from C5 of glucose of dT4k6dG accompanied by epimerization, then proton donation to C5, resulting in a mono-epimerized intermediate; (2) a proton from C3 of glucose is abstracted accompanied by epimerization, followed by proton donation to C3; (3) a ring flip occurs [49]. These reactions need strict stereo control and a cofactor is not required [50]. RmlC and/or RmlC co-complex structures have been obtained with dTDP-phenol, dTDP, dTDP-Glc and dTDP-D-xylose [49,51]. RmlC functions as a homodimer (Figure 5A). The monomer consists of 11 β-strands and seven α-helices that can be divided into three parts, including an N-terminal portion, a core active site, and a C-terminal portion. A His-Asp dyad (Figure 5B) in the active site is crucial in the RmlC catalytic mechanism because a conserved His65 residue from the His-Asp dyad extracts C5 and C3 protons (Figure 5B). Moreover, Tyr134 is essential for epimerization and for proton incorporation at C5. However, a water molecule may replace Tyr134 to facilitate C3 proton incorporation (Figure 5B) [49].

**Figure 6:**
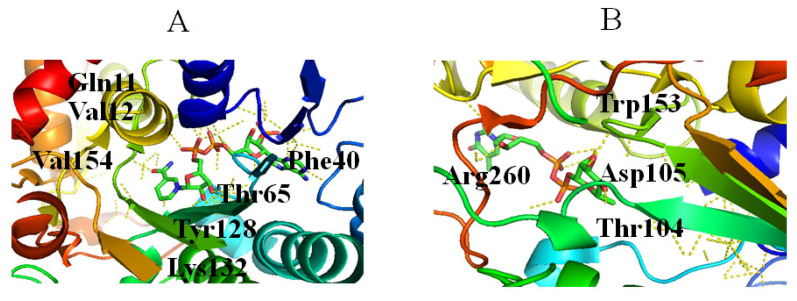
Stereo view of RmlD bound to NADH (PDB entry 1KC3) (**A**) and dTDP-Rha (PDB entry 1KC3) (**B**). Hydrogen bonds are shown as red lines. Helices, sheets, and loops of RmlD are colored blue, purple, and beige, respectively. C, N, O, and P elements of ligands are green, blue, red, and brown, respectively. RmlD is shown in cartoon representation, and ligands are shown as sticks [22].

RmlD catalyzes the last step in the dTDP-Rha biosynthetic pathway, in which the C4 keto group of dTDP-4-keto-Rha is reduced to a hydroxyl group to produce dTDP-Rha (Figure 1) [52,53]. During the reaction, proton transferred from the nicotinamide ring of the cofactor to the C4 keto group requires the assistance of Mg^2+^ [52]. RmlD is a homodimer, and the monomer consists of two domains: an N-terminal domain that binds NAD(H), and a C-terminal domain that binds substrate [52]. Various residues are involved in interactions with NAD(P)H, including (1) a ribose moiety located in the space formed by Ala62, Ala63, Gly7 and Gly10, in which the 2′- and 3′-hydroxyl groups of the ribose ring and Lys132 from the conserved YXXXK motif engage in two hydrogen bonds (Figure 6A); (2) the adenine ring of the cofactor located in a pocket formed by Val31, Asp39, Phe40, Ala62, Ala63, Leu80, and Phe40, in which Asp39 interacts with adenine via hydrogen bonds (Figure 6A); (3) Gln11 and Thr 65 interact with diphosphate (Figure 6A) [52]. Three glutamic acids (Glu15, Glu190, and Glu292) of two monomers bind to Mg^2+^ [52], and dTDP-Rha binds in a pocket of RmlD built from the hydrophobic parts of the side chains of Thr65, Tyr106, Tyr128, and Val67, together with the nicotinamide ring of the cofactor [52]. Additionally, Thr104, 105, Trp153, the carboxamide group of the cofactor, and a water molecule interact with L-Rha (Figure 6B) [52].

### 2.2. Biosynthetic Pathway of GDP-Rha

**Figure 7:**
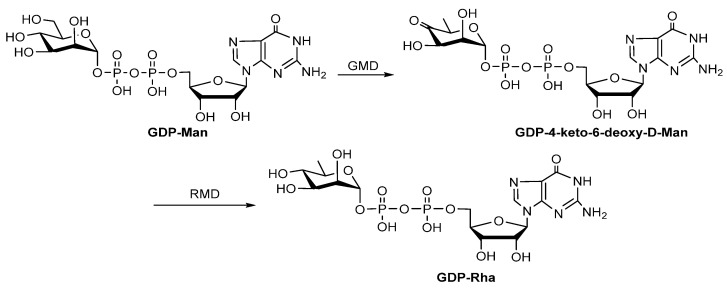
Biosynthesis pathway of GDP-Rha [22].

D-Rha is a rare 6-deoxy monosaccharide found in the LPS of pathogenic bacteria [54]. GDP-Rha is the precursor for the biosynthesis of D-Rha-containing compounds, and it is synthesized in two steps: (1) GDP-mannose-4,6-dehydratase (GMD) catalyzes the conversion of GDP-D-mannose (GDP-Man) to GDP-4-keto-6-deoxy-D-Man; (2) GDP-6-deoxy-D-lyxo-hexos-4-ulose-4-reductase (RMD) catalyzes the production of GDP-Rha (Figure 7). Both GMD and RMD are members of the short-chain dehydrogenase/reductase (SDR) family. GMD is homologous to RmlB, while RMD is homologous to RmlD. However, GMD and RMD cannot catalyze the conversion of dT4k6dG to dTDP-Rha, indicating that enzymes involved in the GDP-D-Rha biosynthesis pathway possess strict substrate specificity. The functions of GMD and RMD from *Aneurinibacillus thermoaerophilus* strain L420-91 (T) [55] and *Pseudomonas aeruginosa* [56] have been confirmed in vitro.

GMD is present in bacteria [57], plants [58], and animals [59], and its production serves as a branch point for several different deoxyhexoses, such as GDP-Rha, GDP-L-fucose, GDP-6-deoxy-D-talose, and the GDP-dideoxy amino sugars [56]. GMD functions as a homodimer [60] or a homotetramer [56] in cells. In particular, PBCV-1 GMD behaves as a bifunctional enzyme, displaying not only dehydratase activity but also a strong NAD(P)H-dependent reductase activity toward GDP-4-keto-6-deoxy-D-Man (the dehydration product), leading to the formation of GDP-Rha [61]. The crystal structures of GMD from *E. coli* [62], *Arabidopsis thaliana* [58], *P. aeruginosa* [56], and *Paramecium bursaria Chlorella virus 1* (PBCV-1) [63] have been reported. The GMD monomer folds into two domains: a N-terminal cofactor-binding domain and a C-terminal substrate-binding domain. Residues of GMDs that contact the GDP moiety are highly conserved, including Val190, Asn179, Lys193, Arg218, Arg279, and Glu282. However, the hexose moiety has not been successfully crystallized. The crystal structure of RMD from *Aneurinibacillus thermoaerophilus* was reported in 2008, but the quality of the crystal structure was not good [64].

### 2.3. Biosynthesis Pathway of UDP-Rha

**Figure 8:**
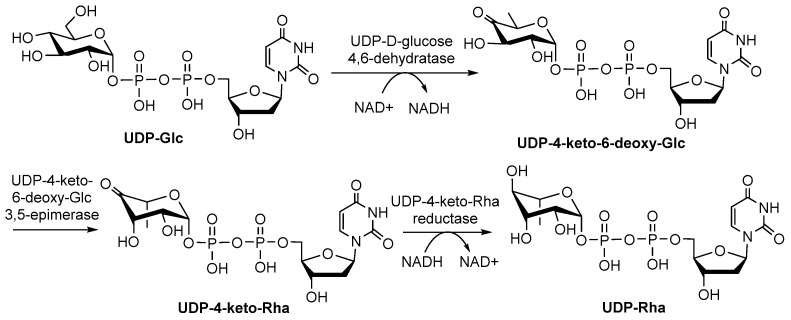
Biosynthesis pathway of UDP-Rha [22].

UDP-Rha is found in fungi and plants, and its biosynthesis pathway involves dehydration, epimerization, and reduction, similar to dTDP-Rha (Figure 8) [9]. Three isoenzymes (UDP-Rha synthases, RHMs) RHM1, RHM2/RHM4 and RHM3 convert UDP-Glc to UDP-Rha via UDP-4-keto-6-deoxy-glucose (U4k6dG) as an intermediate [65]. All the RHMs function as the activities of UDP-D-Glc 4,6-dehydratase, UDP-4-keto-6-deoxy-D-Glc 3,5-epimerase, and UDP-4-keto-L-Rha4-keto-reductase, respectively [66]. Other enzymes, such as a bifunctional enzyme named nucleotide-Rha synthase/epimerase-reductase (NRS/ER), can also act on the intermediate U4k6dG to form UDP-Rha [56,65]. Notably, it is not known what substrates are utilized by Rha-Ts as donors in plants because there are two rhamnose donors in plants (UDP-Rha and dTDP-Rha).

## 3. Rha-Ts Generating RCCs in Bacteria

Glycosyltransferases (GTs) are a large family of enzymes that catalyze the transfer of saccharide moieties from glycosyl donors to a broad range of acceptor substrates, including monosaccharides, oligosaccharides and polysaccharides, lipids, proteins, nucleic acids, and small organic molecules, to form complex carbohydrates and glycoconjugates that are essential to many fundamental biological processes [1]. There are three main methods for the classification of GTs: Firstly, based on the anomeric configuration of reactants and products, GTs are classified as inverting or retaining enzymes; Secondly, GT-A, GT-B, and GT-C topologies of GTs are divided based on Rossmann-fold domains and the locations of donors and acceptors; Thirdly, according to sequence similarity, GTs are divided into 114 different families, as listed in the carbohydrate-active enzymes (CAZy) database (http://www.cazy.org accessed on 14 March 2022). Rha-Ts are GTs that generate RCCs, which are universally present in bacteria [67,68]. However, biochemical knowledge on Rha-Ts is still limited.

### 3.1. Rha-Ts from Geobacillus Stearothermophilus

**Figure 9:**
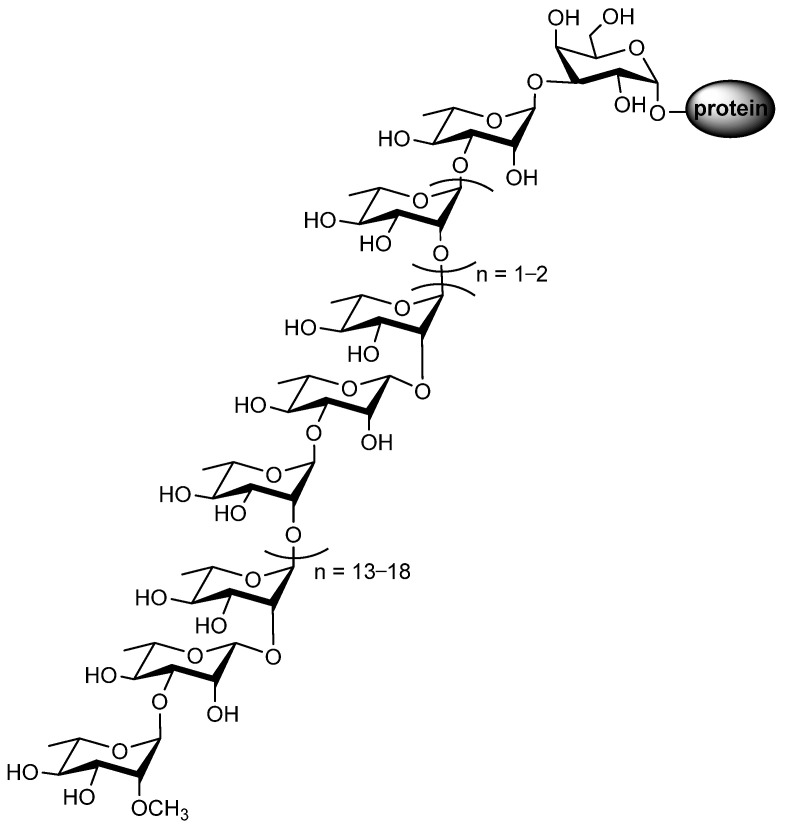
The glycan structure of the *Geobacillus stearothermophilus* S-layer glycoprotein.

The S-layer protein of *Geobacillus stearothermophilus* NRS 2004/3a serves as a model for investigating O-glycosylation pathways in bacteria, and the glycans of this protein are 2-OMe-α-L-Rha-(1→3)-β-L-Rha-(1→2)-α-L-Rha-(1→[2)-α-L-Rha-(1→3)-β-L-Rha-(1→2)-α-L-Rha-(1→]_n=13–18_[2)-α-L-Rha-(1→]_n=1–2_3)-α-L-Rha-(1→3)-β-D-Gal-(1→Protein (Figure 9) [69,70].

**Figure 10:**
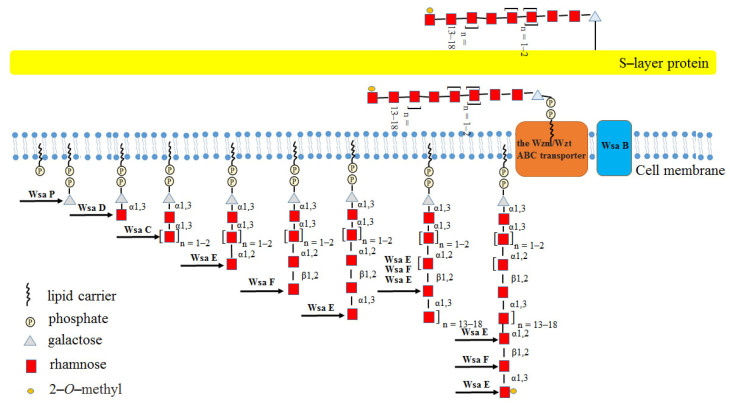
Diagram of the biosynthesis pathway of the S-layer glycoprotein glycan of *Geobacillus stearothermoph ilus* NRS 2004/3a.

The polycistronic S-layer glycosylation (*slg*) gene cluster encodes four GTs, of which three Rha-Ts (WsaC, WsaD and WsaF) catalyze the biosynthesis of the glycan [70]. The biosynthesis pathway for this glycan is initiated by the transfer of a galactose residue to a membrane-associated lipid carrier, followed by two steps catalyzed by α-1,3-Rha-Ts (WsaC and WsaD) that add Rha to build up the [2)-α-L-Rha-(1→]_n=1–2_3)-α-L-Rha-(1→3) linker, and two Rha-Ts (WsaE and WsaF) then extend the glycan chain by adding the repeating trisaccharide motif [→2)-α-L-Rha-(1→3)-β-L-Rha-(1→2)-α-L-Rha-(1→]_n=13–18_ through addition of Rha. The complete glycan chain is thereafter transported across the membrane by an ATP-binding cassette transporter (ABC transporter) and transferred to the S-layer protein by oligosaccharyltransferase WsaB [70]. The functions of WsaC−WsaF were proved by using the chemically synthesized β-D-Gal-(1→O)-octyl as substrate. Among them, both WsaC and WsaD are transmembrane proteins, and the activity of WsaC requires membranes, while WsaD can only recognize the natural substrate; WsaE is a multifunctional enzyme, and the N-terminal domain of WsaE possesses methylase activity, whereas the central and C-terminal domains of WsaE possess Rha-Ts activity, generating α-1,2 and α-1,3 linkages; WsaF is a β-1,2-Rha-T enzyme (Figure 10) [70].

WsaF is a dimer formed by two monomers that consist of two GT-B-fold domains and a cleft between the two domains [69]. dTDP-Rha interacts with WsaF, and the dTDP-WsaF and dTDP-Rha-WsaF complex structures revealed that thymidine contacts with K302 and L303, while V282 and G283 interact with thymidine via van der Waals forces, pyrophosphate binds to G63, R249 and K302 through hydrogen bonds, Rha contacts N227, K225 and E333 directly, and Y329 engages in a stacking interaction with the hydrophobic face of Rha [69]. The crystal structure of the WsaF–acceptor complex has not been reported. However, the acceptor fragments of both α-L-Rha-(1-2)-α-L-Rha-(1-3)-α-L-Rha and α-L-Rha-(1-2)-α-L-Rha-(1-3)-α-L-Rha-(1-3)-α-D-Gal were modeled manually in the tunnel using PyMOL, suggesting that G63, I65, P54, S55, A140, Q170, D171, E173 and F176 would form van der Waals interactions with the acceptor fragments. This was confirmed by mutant studies [69].

### 3.2. Spinosyn SpnG Rha-T from Saccharopolyspora Spinosa

Spinosyn from *S. spinosa* is a type of macrocyclic lactone that has been used as an agricultural antibiotic [71,72]. The entire spinosyn biosynthetic gene cluster contains 19 genes, including 5 large genes (*spnA*, *spnB*, *spnC*, *spnD*, and *spnE*) encoding a type I polyketide synthase, 4 genes (*spnF*, *spnJ*, *spnL*, and *spnM*) encoding proteins involved in intramolecular C-C bond formation, 4 genes (*spnG*, *spnI*, *spnK*, and *spnH*) encoding proteins involved in Rha attachment and methylation, and 6 genes (*spnP*, *spnO*, *spnN*, *spnQ*, *spnR*, and *spnS*) encoding proteins involved in forosamine biosynthesis [72]. SpnG is known to be capable of transferring Rha from dTDP-Rha donors to spinosyn aglycone (AGL) and to display relaxed substrate specificity [73].

SpnG forms a C2-symmetric homodimer, and each monomer contains two domains connected by a long loop (residues 183–209) [71]. The C-terminal domain binds the donor substrate (dTDP-Rha) and the N-terminal domain binds the acceptor substrate [71,73]. Specifically, interactions between dTDP-Rha and SpnG include thymine contacting L279, V277, P257, and L279; α-phosphate forming hydrogen bonds with G296, T297, and T297; β-phosphate making hydrogen bonds with M227 and V228; the 3-OH group of deoxyribose directly forming a hydrogen bond with N202; and Rha contacting with D316, Q317, Y314, and W142 [71]. The crystal structure of a SpnG–acceptor complex has not been reported.

### 3.3. WbbL from Mycobacterium Tuberculosis

The cell wall of *M. tuberculosis*, essential for cell proliferation and growth, is composed of peptidoglycan, arabinogalactan, and mycolic acids [7]. The galactan of arabinogalactan combines with peptidoglycan via a disaccharide linker, α-L-Rha-(1→3)-α-D-GlcNAc-(1→P), to form the integrated mycobacterial cell wall [74]. The Rha-T enzyme WbbL forms the disaccharide linker by transferring Rha from dTDP-Rha to decaprenyldiphosphoryl-α-D-N-acetyl glucosamine (GlcNAc-PP-DP) [75]. The *wbbL* gene is essential for mycobacterial viability and is found in the genomes of all mycobacteria [76]; hence, it is an attractive target for antituberculosis therapeutics. Activity analysis of WbbL was performed using endogenous GlcNAc-PP-DP as a substrate, and a microtiter plate method was established [74]. The bioinformatics analysis of WbbL showed that it belongs to the GT2 family with a fold characteristic of the GT-A superfamily [74], members of which can utilize dTDP-β-Rha as a substrate and produce an α-Rha product. In addition, this protein has a N-terminal GT domain, no signal peptide or transmembrane helices, and it is located outside the membrane.

### 3.4. Rha-Ts from Pseudomonas Aeruginosa

*Pseudomonas aeruginosa* is a pathogen of plants and animals, and an opportunistic human pathogen that causes serious nosocomial infections [77]. LPSs are major virulence factors composed of three distinct regions, i.e., lipid A, core oligosaccharide (OS), and O polysaccharide (O antigen), which contain diverse repeating saccharide units. In this section, we focus on Rha found in OS and O antigens.

OS is divided into two types: one is capped (linked to O polysaccharides) with O antigen through an α-1,3-linked L-Rha, while the other is uncapped (devoid of O polysaccharides) and contains an α-1,6-linked L-Rha [78]. Gene knockout analysis showed that *migA* and *wapR* genes encode α-1,3 Rha-Ts and α-1,6 Rha-Ts, respectively, which are responsible for the biosynthesis of the α-1,3-linked L-Rha and α-1,6-linked L-Rha [78]. O antigen can also be divided into two types: heteropolymeric O antigen (formerly called B band), containing mannosuronic acid derivatives with *N*-acetyl-D-fucosamine (D-FucNAc), and an alternative LPS containing the common polysaccharide antigen (CPA; formerly called A band). There are repeating units of O-polysaccharides of A band, namely (→3-α-D-Rha-(1→2)-α-D-Rha (1→3)-α-D-Rha α-1→), containing a D-Rha moiety [79]. D-Rha-Ts (including WbpY, WbpX and WbpZ) catalyze the transfer of D-Rha to an acceptor [80,81].

**Figure 11:**
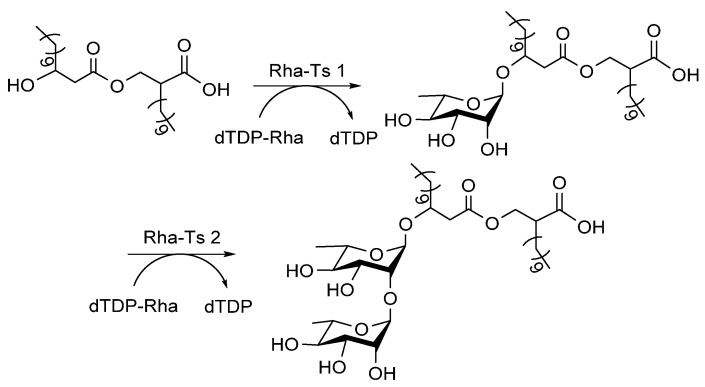
Rha-Ts involved in the biosynthesis of rhamnolipids [22].

Rhamnolipids are detergents composed of α-D-(α-D-hydroxyalkanoyloxy) alkanoic acids (HAA) derivatized with one or two Rha sugars (monorhamnolipids and dirhamnolipids; Figure 11), which are secreted by *P. aeruginosa* [82]. Rha-Ts I [83] and Rha-Ts II [84] generate rhamnolipids, and their mechanism has been determined: (1) Rha-Ts I are encoded by *rhlA* and *rhlB* genes, and gene knockout analysis of these genes indicated that RhlA forms HAA, while RhlB is a Rha-T enzyme [85], and the heterologous expression of RhlA and RhlB was achieved [86]; (2) gene knock-in assay proved that RhlC encodes Rha-Ts II, which transfers the second Rha to dirhamnolipids [84].

Additionally, a Rha-T EarP derived from *P. aeruginosa* has been reported that transfers Rha from dTDP-Rha to Arg32 of the translation elongation factor P (EF-P) [87,88]. This rhamnosylation of Arg32 by EarP can activate the functions of EF-P, which is important in the process of protein translation in ribosome. EarP is also discovered in other clinically relevant bacteria [89,90], indicating that this type of post-translational modification strategy is crucial for protein translation and bacteria pathogenicity [87,88].

### 3.5. Rha-Ts from Streptococcus Pneumoniae

Capsular polysaccharides (CPSs) are produced by almost all isolates of *S. pneumoniae* recovered from cases of invasive disease, and they are major virulence factors and immunogens [91]. Rha-containing CPS has been identified in at least 27 serotypes. Rha-containing CPS of *S. pneumoniae* is particularly attractive: (1) L-Rha may increase the immunogenicity of CPS based on the immune analysis of 23F CPS, showing that α-(1→2)-linked L-Rha is a dominant antigen [92]; (2) modified L-Rha may increase the stability of CPS based on the analysis of a 19F CPS analog in which a residue of carba-L-Rha was inserted into the natural trisaccharide, and this increased the stability of CPS [93]; (3) Rha-Ts are the most prevalent GT genes in *S. pneumoniae* cps loci [94]. Therefore, studies on Rha of *S. pneumonia* CPS may provide a new strategy for developing novel drugs to treat anti-pneumococcal infections. However, new serotypes should be identified, and attempts to determine the structures of CPSs and Rha-Ts have been reported [95,96].

### 3.6. Rha-Tss from Other Bacteria

Although numerous Rha-Ts have been predicted, the in vitro biochemical knowledge of these enzymes is limited. Gene mutants have confirmed the functions of some Rha-Ts, including RgpF [97], WbgA [97], AceR [98], AntB [99], and GacB [100]. Heterologous expression has also been used to confirm the functions of Rha-Ts, as exemplified by HlpA/RtfA [101]. Rha-Ts from *Mycobacterium smegmatis* [93], *Streptococcus anginosus* [102], serotype VIII capsular polysaccharide (CPS) of Group B Streptococci (GBS) [103], and *Vibrio cholera* [104] have also been reported.

This review mainly overviews the research advance of the Rha-Ts derived from bacterial; however, there are also other enzymes involved in RCC biosynthesis that will not be described in detail here. For example, several Rha-Ts from plants have been reported [105,106,107]. Additionally, in recent years, α-L-rhamnosidase has been found to synthesize RCCs by a reverse hydrolyzing mechanism, which has attracted extensive attention [108,109].

## 4. Application of RCC in the Research and Development of New Drugs

### 4.1. Rha Increases the Immunogenicity of Tumour-Associated Carbohydrate Antigen (TACA) Vaccines

TACAs are carbohydrates expressed at high levels on the surface of tumor cells [110,111], and anti-TACA vaccines have been well developed [112]. However, the immunogenicity of TACAs is very low [113]. Saccharide conjugating to proteins can increase its immunogenicity, and this approach was then widely applied in conjugation vaccinations [114]. Although some glycoconjugate TACA cancer vaccines have shown promising therapeutic potential, no vaccine has yet achieved a satisfactory survival rate in clinical trials [115,116]. Guo group developed both positive and negative immunotherapies with unnatural TACAs for testing against cancers [117,118]. However, the quality control of reactions was difficult, and unexpected immune responses to proteins and linkages limited their application.

To solve these problems, two strategies have been developed: using a low-molecular-weight peptide (such as YAF) in place of proteins to increase immunogenicity of TACAs, and antigens targeting antigen-presenting cells (APCs) [117,118]. Additionally, saccharide binding to Rha can improve immunogenicity, as demonstrated by Oyelaran et al. who reported that human serum contains high levels of anti-Rha antibody [119]. Zhang et al. reported that L-Rha conjugated with truncated MAGE-A3 enhanced the immunogenicity of melanoma-associated antigen A3, thereby stimulating antitumor immune responses [120]. A study by Sarkar et al. showed that L-Rha binding to carbohydrate antigens enhanced antigenicity in mice [121]. In 2013, this team also successfully formulated a MUC1 VNTR TACA conjugate into a liposome-based anticancer vaccine, and the immunogenicity of the vaccine was further augmented by incorporating surface-displayed L-Rha epitopes onto liposomes [122]. Li et al. reported a strategy targeting tumor cells using ligand-incorporated Rha-functionalized liposomes [123]. Additionally, L-Rha epitopes can also enhance cellular immunogenicity. Partha et al. reported that the Rha-decorated liposomal Pam_3_Cys-MUC_1_-Tn vaccine showed higher cellular immunogenicity [2]. In addition, the immunogenicity of Rha-decorated liposomal Pam_3_Cys-MUC_1_-Tn was further augmented in mice when received human anti-Rha antibodies prior to its vaccination [124]. Additionally, Rha and sTn antigen, co-conjugated to bovine serum albumin (BSA), significantly enhanced antigen uptake through the involvement of Rha-specific antibodies [125]. Together, these studies showed that TACA vaccines containing Rha can increase immunogenicity. Compared with Galα1-3Galβ1-4GlcNAc-R (α-Gal epitope), Rha not only increases the immunogenicity of TACAs, but also can be used directly in wild-type mice [126].

In addition to TACA vaccines, the strategies of enhancing the monoclonal antibodies’ (mAbs) efficacy were also developed by using high levels of anti-Rha antibody of the human serum [127,128]. MAbs are one of the most rapidly growing drug classes used for the clinical practice, such as cancer and infectious and autoimmune diseases. Complement-dependent cytotoxicity (CDC) and antibody-dependent cell-mediated cytotoxicity (ADCC) are effector functions for antibodies to deplete target cells [128]. Rituximab is one of the commercially available mAbs, which is site-specifically conjugated with the Rha hapten to generate rituximab–Rha conjugates, to recruit anti-Rha antibodies onto the cancer cell surface and further form an immune complex that leads to magnifying ADCC and CDC simultaneously [128]. Ou et al. reported an efficient chemoenzymatic synthesis of structurally well-defined conjugates of antibody–rhamnose clusters to recruit natural anti-rhamnose antibodies for the enhancement of the CDC effects [127]. In addition, Coen et al. reported on antibody-recruiting glycopolymers (ARGPs) that consist of polymeric copies of a rhamnose motif, which can bind anti-Rha antibody of the human serum, for the design of potent immunotherapeutics that mark target cells for destruction by the immune system through ADCC [129]. These studies developed general and cost-effective approaches to augment the mAb effector functions with the engagement of anti-Rha antibody of the human serum that may have broad applications.

### 4.2. Rha-Containing Tumor-Killing Agents

Many natural products are known to have human health benefits, such as saponins and tumor-killing agents. The relationships between biological activity and chemical structure of some tumor-killing agents indicate that Rha may play a crucial role in determining biological properties. For example, kaempferol-3-O-(3″,4″-di-O-acetyl-α-L-rhamnopyranoside; SL0101) from *Forsteronia refracta* can inhibit the activity of Ser/Thr protein kinases (RSKs) that are closely related to the proliferation and metastasis of many tumor cells [130]. During this process, acylation of the Rha moiety of SL0101 is required for high-affinity binding and selectivity [118]. In addition, the Rha moiety of solamargine and solasonine is a key factor in anticancer activity [131,132]. Lou group demonstrated why Rha plays an important role in the anticancer activity of solasodine-derived rhamnosides; they reported that Rha-binding lectins (RBLs) on the surface of tumor cells conjugated with Rha to mediate the transportation of rhamnosides [133].

Furthermore, due to specificity of the interactions between carbohydrates and cell receptors, a lectin-directed enzyme-activated prodrug therapy (LEAPT) strategy was developed [134]. Specifically, in the first phase of this strategy, a glycosylated enzyme is targeted to specific cell types or tissues; in the second phase, prodrugs capped with sugars are administered; the glycosylated enzyme is then able to activate the prodrugs at the site of interest by cleaving the prodrug linkage; the interaction of both prodrug and enzyme relies on their precise glycosylation, and Rha-doxorubicin and Rha-5-fluorouracil are effective examples [134]. Although the Rha of tumor-killing agents is a key factor in tumor killing, L-Rha cannot kill tumor cells directly because it does not affect energy metabolism [135].

### 4.3. Inhibitors of Rha Synthetases as Drug Targets

Many prevalent and opportunistic pathogens, including *M. tuberculosis*, *P. aeruginosa*, and *S. pneumoniae*, are particularly difficult to treat due to their intrinsic chemo-resistance and their ability to acquire further resistance mechanisms against antimicrobial agents. Rha biosynthesis pathways have been discovered in numerous bacteria and fungi, but they have not been discovered in humans, hence they might be potential therapeutic targets [136,137]. The first nanomolar inhibitors of RmlA from *P. aeruginosa* were thymine analogs, and some inhibitors also showed inhibitory activity against *M. tuberculosis* [138]. In addition, L-Rha-1-C-phosphonate is the best inhibitor of Cps2L, and a fluorine atom at C1 can increase inhibition by 25%, but two fluorine atoms at C1 had an adverse effect [139]. Furthermore, RmlC is the most promising therapeutic target because it possesses high substrate specificity and it does not require a cofactor [140].

## 5. Conclusions

RCCs are present in bacteria but not in humans and other mammals, making them valuable for tumor immunotherapy and treating antibacterial infections. To date, RCCs have been studied extensively, and produced a series of excellent results, i.e., the discovery of the biosynthetic pathways of three rhamnose donors, the discovery of Rha-Ts, and their application to the treatment of various diseases. In this review, the biosynthesis pathways and the properties of the related enzymes from three donor substrates, including dTDP-Rha, GDP-Rha, and UDP-Rha, were reviewed in detail, which is of great significance for the development of the strategies for the preparation of donor substrates of Rha-Ts in vitro. In addition, the functions and properties of Rha-Ts were also reviewed, which provides theoretical guidance for the development of Rha-Ts and the enzymatic synthesis of RCCs. It is important to note the complex and diverse structures of the receptor substrates of Rha-Ts, which need to be further studied. However, the research of the synthesis pathways of RCCs from different cells, the properties of related enzymes and their catalytic mechanisms is rather little; therefore, further studies on the biosynthesis and applications of RCCs are being carried out at present and subsequently via the latest biochemical technologies, such as molecular biology, structural biology, and computational biochemistry techniques.

## Supplementary Materials

The following supporting information can be downloaded at: https://www.mdpi.com/article/10.3390/molecules27165315/s1, Table S1: Sugar-1-phosphate substrates of RmlA and their conversion into corresponding NDP-sugars.
