# Supplementary material for: Rhamnose-Containing Compounds: Biosynthesis and Applications
Source: Molecules. 2022 Aug 20;27(16):5315. doi: 10.3390/molecules27165315 (PMC9415975; doi:10.3390/molecules27165315)
Supplement: Supplementary file 1 [file molecules-27-05315-s001.zip › molecules-1833400-supplementary.pdf]

Supplementary information

for

Rhamnose-Containing Compounds: Biosynthesis and Applications

Siqiang Li [1,2], Fujia Chen [1,2], Yun Li [1,2], Lizhen Wang [3], Hongyan Li [1], Guofeng Gu [4,*], Enzhong Li [1,2,*]

1. School of Biological and Food Processing Engineering, Huanghuai University, Zhumadian, 463000, China
2. Institute of Agricultural Products Fermentation Engineering and Application, Huanghuai University, Zhumadian, 463000, China
3. Biology Institute, Qilu University of Technology (Shandong Academy of Sciences), Jinan 250100, China
4. National Glycoengineering Research Center, Shandong Key Laboratory of Carbohydrate Chemistry and Glycobiology, Shandong University, 72 Binhai Road, Qingdao 266237, China

* Correspondence: guofenggu@sdu.edu.cn (G.G.); enzhongli@163.com (E.L.)

Table of Contents

Supplementary Table. S1……………………………………….…………………………………………………Page S3–S5

Table S1. Sugar-1-phosphate substrates of RmlA and their conversion into corresponding NDP-sugars[22]

NO.	Sugar-1-P	Conversion （%）								ref
		dTTP	UTP	GTP	CTP	ATP	dGTP	dCTP	dATP	
**Glc-1**	α-D-glucose-1-phosphate	99	99	80	70	70	+	+	+	[35]
**Glc-2**	2-deoxy-α-D-glucose-1-phosphate	25	22	ND	ND	ND	ND	ND	ND	[26]
**Glc-3**	3-deoxy-α-D-glucose-1-phosphate	96	6.5	+ m3	ND	ND	ND	ND	ND	[26, 37]
**Glc-4**	4-deoxy-α-D-glucose-1-phosphate	98	99	+ m3	ND	ND	ND	ND	ND	[26, 37]
**Glc-5**	6-deoxy-α-D-glucose-1-phosphate	98	99	59.6m3	+	+	+	+	+	[35]
**Glc-6**	4,6-dideoxy-α-D-glucose-1-phosphate	99	ND	9.9m3	ND	ND	ND	ND	ND	[38]
**Glc-7**	2-amino-2-deoxy-α-D-glucose-1-phosphate	99	99	+	40m1	8	+	+	+	[30, 32]
**Glc-8**	3-amino-3-deoxy-α-D-glucose-1-phosphate	99	78	+m3	ND	ND	ND	ND	ND	[30, 32]
**Glc-9**	4-amino-4-deoxy-α-D-glucose-1-phosphate	99	90	ND	ND	ND	ND	ND	ND	[30]
**Glc-10**	6-amino-6-deoxy-α-D-glucose-1-phosphate	99	15	+	+	+	+	+	+	[30]
**Glc-11**	4-amino-4,6-dideoxy-α-D-glucosamine-1-phosphate	99	15	+m3	ND	ND	ND	ND	ND	[30]
**Glc-12**	3-amino-3,4,6-trideoxy-α-D-glucose-1-phosphate	99	ND	ND	ND	ND	ND	ND	ND	[38]
**Glc-13**	3-amino-3,6-dideoxy-α-D-glucose-1-phosphate	10	+	ND	ND	ND	ND	ND	ND	[38]
**Glc-14**	4-amino-2,4-dideoxy-α-D-glucosamine-1-phosphate	ND	ND	+m3	ND	ND	ND	ND	ND	[37]
**Glc-15**	2-acetamido-2-deoxy-α-D-glucose-1-phosphate	50	50	ND	+	30	+	+	+	[25, 30]
**Glc-16**	3-acetamido-3-deoxy-α-D-glucose-1-phosphate	70	2.5	ND	ND	ND	ND	ND	ND	[30, 38]
**Glc-17**	3-acetamino-3,4,6-trideoxy-α-D-glucose-1-phosphate	70	3	ND	ND	ND	ND	ND	ND	[30, 38]
**Glc-18**	4-acetamido-4-deoxy-α-D-glucose-1-phosphate	NA	NA	ND	ND	ND	ND	ND	ND	[30]
**Glc-19**	6-acetamido-6-deoxy-α-D-glucose-1-phosphate	2.5	20	ND	ND	ND	ND	ND	ND	[30]

**Glc-20**	3-acetamino-3,6-dideoxy-α-D-glucose-1-phosphate	99[a]	ND	ND	ND	ND	ND	ND	ND	[38]
**Glc-21**	3-O-Methyl-α-D-glucopyranosyl phosphate	98	97	+[m3]	+	+	+	+	+	[33]
**Glc-22**	3-N,N-dimethyl-3-deoxy-α-D-glucose-1-phosphate	NA	ND	ND	ND	ND	ND	ND	ND	[38]
**Glc-23**	3-azido-3-deoxy-α-D-glucose-1-phosphate	100	22	+	NA	+	+	+	+	[32]
**Glc-24**	4-azido-4-deoxy-α-D-glucose-1-phosphate	+	+	+	+	+	+	+	+	[35, 37]
**Glc-25**	6-azido-6-deoxy-α-D-glucose-1-phosphate	+	+	+[m3]	ND	ND	ND	ND	ND	[37, 41]
**Glc-26**	3-deoxy-3-thio-α-D-glucose-1-phosphate	+	ND	ND	ND	ND	ND	ND	ND	[26]
**Glc-27**	4-deoxy-3-thio-α-D-glucose-1-phosphate	+	ND	ND	ND	ND	ND	ND	ND	[26]
**Glc-28**	6-deoxy-3-thio-α-D-glucose-1-phosphate	+	+	ND	ND	ND	ND	ND	ND	[40]
**Glc-29**	α-D-glucuronic acid-1-phosphate derives	88[m4]	ND	ND	ND	ND	ND	ND	ND	[25]
**Glc-30**	3-O-Butyl-α-D-glucopyranosyl phosphate	99	50	NA	ND	ND	ND	ND	ND	[33]
**Glc-31**	3-O-Hexyl-α-D-glucopyranosyl phosphate	+	+	NA	ND	ND	ND	ND	ND	[33]
**Glc-32**	3-O-Octyl-α-D-glucopyranosyl phosphate	99	27	NA	ND	ND	ND	ND	ND	[33]

**Glc-33**	3-O-Dodecyl-α-D-glucopyranosyl phosphate	97	73	NA	ND	ND	ND	ND	ND	[33]
**Glc-34**	3-O-Hexadecyl-α-D-glucopyranosyl phosphate	32	7	NA	ND	ND	ND	ND	ND	[33]
**Glc-35**	3-O-(2-Methylpropyl)-α-D-glucopyranosyl phosphate	98	51	NA	ND	ND	ND	ND	ND	[33]
**Glc-36**	3-O-(2-Ethylbutyl)-α-D-glucopyranosyl phosphate	96	39	NA	ND	ND	ND	ND	ND	[33]
**Glc-37**	D-glucofuranose-1-phosphate	7	ND	ND	ND	ND	ND	ND	ND	[42]
**Glc-38**	L-glucose-1-phosphate derives	5	NA	ND	ND	ND	ND	ND	ND	[26]
**Glc-39**	6-deoxy-4-keto-α-D-glucose-1-phosphate	+	+	ND	ND	ND	ND	ND	ND	[26]
**Glc-40**	C-(1-Deoxy-a-D-glucopyranosyl) methane phosphonate	97	72	18	18	20	ND	ND	ND	[42]
**Glc-41**	glucose ethylphosphonate	0	ND	ND	ND	ND	ND	ND	ND	[25]
**Fuc-1**	β-L-fucose-1-phosphate	98	NA	NA	NA	NA	ND	ND	ND	[31]
**Fuc-2**	α-L-fucose-1-phosphate	+	ND	ND	ND	ND	ND	ND	ND	[28]
**Fuc-3**	D-fucofuranose-1-phosphate	41	ND	ND	ND	ND	ND	ND	ND	[42]
**Ara-1**	L-arabinofuranose-1-phosphate	58	ND	ND	ND	ND	ND	ND	ND	[42]
**Gal-1**	C-(1-Deoxy-a-D-galactopyranosyl) methane phosphonate	45	2	ND	ND	ND	ND	ND	ND	[42]
**Gal-2**	6-deoxy-6-fluoro-D-galactofuranose-1-phosphate	19	ND	ND	ND	ND	ND	ND	ND	[34]
**Gal-3**	D-galactose-1-phosphate	57	32	ND	ND	ND	ND	ND	ND	[26]
		40	ND	ND	ND	ND	ND	ND	ND	[29]

**Gal-4**	N-acetyl-a-D-galactosamine-1-phosphate	+	ND	ND	ND	ND	ND	ND	ND	[39]
**Gal-5**	C-(1-Deoxy-a-D-glucopyranosyl) methane phosphonate	97	72	18	18	20	ND	ND	ND	[26]
**Xyl-1**	α-D-xylose-1-phosphate	ND	ND	46^{m3}	ND	ND	ND	ND	ND	[37]
**All-1**	a-D-allose-1-phosphate	53^{m2}	36^{m2}	ND	ND	ND	ND	ND	ND	[25, 26]
**Man-1**	α-D-mannose-1-phosphate	99	98	45^{m1}	0^{m1}	8^{m1}	ND	ND	ND	[26, 31, 42]
**Tal-1**	α-D-talose-1-phosphate	99^{m2}	77^{m2}	ND	ND	ND	ND	ND	ND	[27]
**Gul-1**	α-D-gulopyranosyl phosphate	＜6%	＜6%	ND	ND	ND	ND	ND	ND	[27]
**Ino-1**	myo-Inositol-2-phosphate	18	NA	ND	ND	ND	ND	ND	ND	[25]
**Ido-1**	α-D-idopyranosyl phosphate	29^{m2}	＜6%^{m2}	ND	ND	ND	ND	ND	ND	[27]

^{m1}RmlA Q24S, ^{m2} RmlA L89T, ^{m3} RmlA Q83D, ^{m4}RmlA W224H; +: product detected but conversion not reported; NA: no reaction observed; ND: not determined.
